# Differences of IL-1*β* Receptors Expression by Immunocompetent Cells Subsets in Rheumatoid Arthritis

**DOI:** 10.1155/2015/948393

**Published:** 2015-09-10

**Authors:** Alina A. Alshevskaya, Julia A. Lopatnikova, Nadezhda S. Shkaruba, Oksana A. Chumasova, Aleksey E. Sizikov, Aleksander V. Karaulov, Vladimir A. Kozlov, Sergey V. Sennikov

**Affiliations:** ^1^Federal State Budgetary Scientific Institution “Research Institute of Fundamental and Clinical Immunology”, Yadrintsevskaya Street 14, Novosibirsk 630099, Russia; ^2^I.M. Sechenov First Moscow State Medical University of the Ministry of Health, Bolshaya Pirogovskaya Street 2-4, Moscow 119991, Russia

## Abstract

IL-1*β* is involved in the induction and maintenance of chronic inflammation in rheumatoid arthritis (RA). Its activity is regulated and induced by soluble and membrane-bound receptors, respectively. The effectiveness of the cytokine depends not only on the percentage of receptor-positive cells in an immunocompetent subset but also on the density of receptor expression. The objective of this study was to investigate the expression of IL-1*β* membrane-bound receptors (IL-1R1 and IL-1R2) in terms of the percentage of receptor-positive cells and the number of receptors per cell in different subsets of immune cells in RA patients before and after a course of basic (excluding anticytokine) therapy and in healthy individuals. The resulting data indicate differences in the expression of IL-1*β* receptors among T cells, B cells, and monocytes in healthy volunteers and in rheumatoid arthritis patients. The importance of determining both the relative percentage of cells expressing receptors to immunomodulatory cytokines and the number of membrane-bound receptors per cell is highlighted by evidence of unidirectional or multidirectional changing of these parameters according to cell subset and health status.

## 1. Introduction

Rheumatoid arthritis (RA) is characterized by chronic systemic autoimmune inflammation of the connective tissue and is mostly accompanied by lesions in peripheral joints, erosion and degenerative changes in joints, and joint ankylosis [1]. A broad range of cells, from monocyte/macrophage subsets to T and B cells, are involved in the pathogenesis of RA [2–6]. Cytokines, the main mediators of intercellular communication, play a crucial role at all stages of the development of immune responses in this disease. As a broad-spectrum proinflammatory cytokine, interleukin-1*β* (IL-1*β*) is involved in the development of both local and systemic inflammation in rheumatoid arthritis [7, 8]. Its hypersecretion destroys the bone tissue at the local level and also results in a significant disruption of hemodynamics at the systemic level.

IL-1*β* implements its biological effects after binding specific membrane-bound receptors. There are two types of membrane-bound IL-1*β* receptors (IL-1R1 and IL-1R2); however, IL-1R2 does not contain a full-fledged cytoplasmic domain. Hence, IL-1R2 cannot transmit signals to cells and thus acts as a decoy receptor [9, 10]. Therefore, IL-1*β* produces its biological activity against cells only through type 1 receptor [11, 12]; however, the signaling pathways can be initiated only if the interleukin-1 receptor accessory protein (IL1RAcP) is present in the receptor-cytokine complex. Furthermore, cytokine activity is also regulated by the circulating interleukin-1 receptor antagonist (raIL-1) that competes for binding to receptors, thus acting as a specific IL-1 inhibitor [13, 14]. Soluble IL-1 receptors are the extracellular domains of membrane-bound IL-1 receptors [15, 16], which are formed either via proteolytic cleavage catalyzed by specific metalloproteinases [17, 18] or by alternative splicing (so far demonstrated only for type 2 receptors) [19]. The key function of soluble IL-1 receptors is inhibiting the biological effects of cytokines by competing with membrane-bound receptors for binding to the ligand [15, 20]. Additionally, soluble type 1 receptors were shown to have a buffering function for IL-1 ligands [21].

The modulation of cytokine activity depends on a number of parameters, including the levels of soluble mediators, percentage of receptor-carrying cells, ratios between subpopulations by percentage or density of receptor expression, and the ratio between types 1 and 2 receptors per cell. However, simultaneous comprehensive investigation and comparison of the various components of the IL-1*β* receptor apparatus in rheumatoid arthritis using both qualitative (counting the percentage of IL-1R1^+^ and IL-1R2^+^ cells) and accurate quantitative characteristics (density of receptor expression on cell surface) have not been performed. Hence, our study aimed to investigate the changes in expression of IL-1*β* receptors in rheumatoid arthritis.

## 2. Materials and Methods

### 2.1. Patients and Samples

The group of patients with RA consisted of 40 persons who were hospitalized at the Clinic of Immunopathology under the Federal State Budgetary Scientific Institution “Research Institute of Fundamental and Clinical Immunology,” in Novosibirsk, Russia. Diagnosis of RA was verified in accordance with the ACR (American College of Rheumatology) criteria (2010). The severity of RA was determined by counting the number of painful and swollen joints among 28 specified joints, determination of the erythrocyte sedimentation rate, assessment of each patient's general well-being according to the Visual Analogue Scale (range 0–100 mm), and subsequent calculation of the DAS28 index. At the time of their admission to the clinic, all patients had a high disease activity (DAS28 > 5.9). Blood samples were collected from each patient during the acute stage (*n* = 40, aged 28–75 years, six men and 34 women) and after effective course of treatment (*n* = 21, aged 28–75 years, two men and 19 women) that involved besides basic methotrexate therapy (15–20 mg in week) either biological agents (Rituximab, 500 mg intravenous in first and 15th days, 7 women) or methylprednisolone (intravenous pulse therapy, 500 mg for three days, two men and 12 women) one day before discharge from a hospital. None of the 23 investigated parameters was shown to have significant differences between patients who received different types of therapy. Additionally, there were no significant differences between men and women and between patients of different age groups, so the data presented were not divided by therapy or age. In all cases, therapeutic efficacy was assessed using the criteria established by the European League against Rheumatism and revealed positive clinical or laboratory dynamics: an alteration of DAS28 by >1.2 from the initial value was used to classify patients as having responded to therapy.

We obtained human peripheral blood mononuclear cells (PBMC) from 150 healthy individuals (aged 18–59 years, 67 men and 83 women) from the Novosibirsk Blood Center. The status of the donors was determined through a questionnaire and a latex test for C-Reactive Protein (CRP; LLC Olvex diagnosticum, Russia). Individuals with serum CRP concentration <6 mg/mL were included as donors. We sampled peripheral blood only after obtaining informed consent from all donors and patients. All work was conducted in accordance with the Declaration of Helsinki (1964). The study was approved by the local ethics committee of the Federal State Budgetary Scientific Institution “Research Institute of Fundamental and Clinical Immunology” (protocol number 52, 23.06.2010).

### 2.2. Isolation of Peripheral Blood Mononuclear Cells

We isolated PBMCs from whole blood by Ficoll (Pharmacia Fine Chemicals, Uppsala, Sweden) density centrifugation (*ρ* = 1.077 g/L) as previously described [22]. Cells were cultured in 96-well plates in RPMI-1640 medium (Biolot, Russia) supplemented with 10% fetal calf serum, 2 mM L-glutamine, 10 mM HEPES buffer, 0.5 mM 2-mercaptoethanol, 80 mg/mL gentamicin, and 100 mg/mL benzylpenicillin for 24 h at 37°C with 5% CO_2_ in the presence or absence of LPS (055:B5; Sigma-Aldrich, St. Louis, MO, USA) at a final concentration of 200 ng/mL.

### 2.3. Flow Cytometry

We identified subsets of mononuclear cells by immunophenotyping using human-specific fluorescently labeled mouse monoclonal antibodies: allophycocyanin- (APC-) conjugated anti-CD3 (clone OKT3), fluorescein isothiocyanate- (FITC-) conjugated anti-CD14 (clone 61D3) and phycoerythrin-cyanine 7 (PE-Cy7) conjugated anti-CD19 (clone HIB19) (eBioscience, San Diego, CA, USA), anti-human IL-1R1, and anti-human IL-1R2 PE (R&D Systems, Minneapolis, MN, USA). BD QuantiBRITE PE beads (BD Biosciences, San Jose, CA, USA) were used to convert cell fluorescence data of positive cells into the absolute number of receptors per cell [23].

### 2.4. Determination of IL-1*β*, raIL-1, and Soluble IL-1 Receptor Concentration in Serum

We evaluated protein levels in peripheral blood serum using ELISA kits for sIL-1R1, sIL-1R2 (RayBiotech, Norcross, GA, USA), IL-1*β*, and raIL-1 (JSC Vector-Best, Novosibirsk, Russia), as per the manufacturer's instructions.

### 2.5. Statistics

We analyzed statistical data with STATISTICA 7.0 software (StatSoft, Tulsa, OK, USA). Independent samples were tested for statistical significance using the Mann-Whitney test and nonparametric Kruskal-Wallis analysis of variance by rank and median multiple comparisons. The data are expressed as the median and interquartile range. We evaluated correlations among parameters with the Pearson test (*p* < 0.05). A *p* value of *p* < 0.05 was considered statistically significant.

## 3. Results

### 3.1. Expression of IL-1*β* Receptors Types 1 and 2 on Subpopulations of Mononuclear Cells

**Figure 1:**
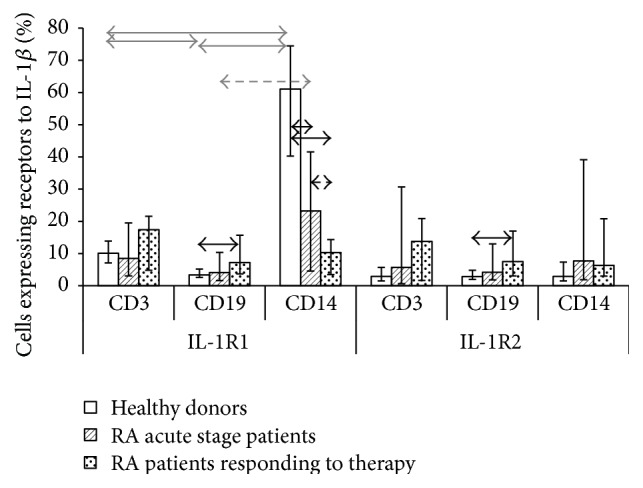
Percentage of cells expressing IL-1R1 and IL-1R2 in PBMC populations derived from healthy individuals (*n* = 15) and RA patients in the acute phase (*n* = 33) and after response to treatment (*n* = 21). The data are presented as the median and interquartile range. The arrows denote significance of intergroup differences, *p* < 0.05: black ones, between the studied groups; grey ones, between the subsets.

**Figure 2:**
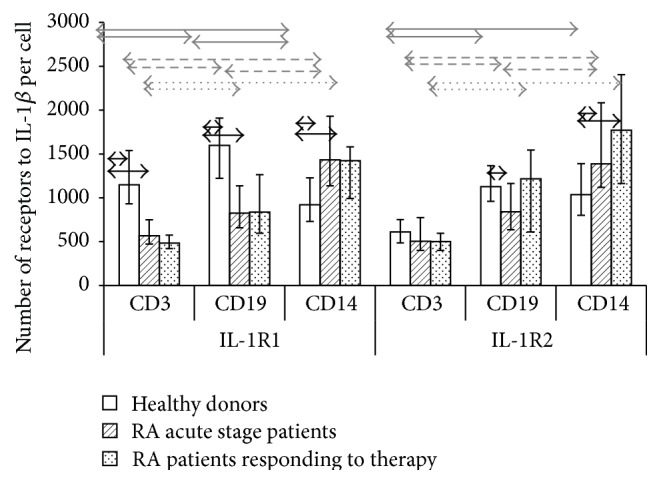
Density of IL-1R1 and IL-1R2 expression on cells in mononuclear cell subpopulations derived from healthy individuals (*n* = 150) and RA patients in the acute phase (*n* = 33) and after response to treatment (*n* = 21). The data are presented as the median and interquartile range. The arrows denote significance of intergroup differences, *p* < 0.05: black ones, between the studied groups; grey ones, between the subsets.

To study expression of membrane-bound receptors of the proinflammatory cytokine IL-1*β*, which plays a key role in both the development and severity of the pathological process in RA, one needs to assess expression of types 1 and 2 receptors on cells involved in the pathology. The results of previous studies involving healthy individuals [23] demonstrate that there are differences both in the number of cells expressing IL-1 receptors types 1 and 2 and in the number of membrane-bound receptors on subpopulations of peripheral blood mononuclear cells (PBMC). However, the high percentage of receptor-expressing cells in subpopulations does not always associate with high receptor level, and* vice versa*. We selected three main subpopulations of peripheral blood immunocompetent cells: T cells (CD3^+^), B cells (CD19^+^), and monocytes (CD14^+^), because they are most actively involved in the systemic inflammatory response and also take part in local responses. We tested PBMCs derived from RA patients during the acute disease stage and after they had responded to therapy to study the features of the pathogenic process and the reaction of cytokine receptors expression to treatment. The percentage of cells carrying types 1 and 2 receptors was assessed in each subpopulation (Figure 1) and the number of membrane-bound receptors on cells was calculated (Figure 2).

The distribution of receptors on cell subpopulations was found to differ between RA patients and healthy individuals. Whereas in healthy individuals, monocytes had the highest percentage of cells expressing type 1 receptors, the density of expression of these receptors was the lowest. In contrast, in RA patients in the acute phase, the percentage of CD14^+^ cells expressing IL-1R was much lower, while the density of expression was significantly higher compared both with healthy volunteers and with the other cell subsets studied. In terms of expression of IL receptors types 1 and 2, consistent differences in percentage of positive cells between T cell, B cell, and monocyte subpopulations were detected neither in healthy individuals nor in RA patients regardless of the disease phase. However, in terms of density of expression of these receptors, T cells were characterized by the lowest number of receptors compared with B cells and monocytes in all the studied groups, while the greatest number of receptors was detected on monocytes in RA patients, both in the acute phase and in patients who showed a clinical response to treatment.

It was demonstrated that the percentage of monocytes expressing type 1 receptors was consistently decreased in RA patients who showed a clinical response to treatment, while the density of expression of these receptors on monocytes remained unchanged.

Thus, it was found that B cells and monocytes showed oppositely directed changes in percentage of IL-1R1-positive cells and the number of receptors on these cells in RA patients compared with normal controls, but the change in IL-1R1 on B cells in RA patients correlated with the change on monocytes (as confirmed by its positive correlation with *r* = 0.66, *p* < 0.05, for both indicators during the acute phase of the disease and *r* = 0.71 and *r* = 0.48 for the percentage of positive cells, resp., in patients who had responded to treatment) but in an opposite direction. This indicates that there are different possible mechanisms for changing the common pool of membrane-bound receptors in a subpopulation and there is an interrelationship between indicators of receptor expression for different subpopulations of immunocompetent cells.

It was found by comparing the expression parameters of different types of receptors that both the percentage of positive cells and the number of receptors per cell in healthy individuals significantly differ for IL-1R1 and IL-1R2 in each subpopulation studied. RA patients do not exhibit these differences, regardless of the phase of the disease.

### 3.2. Expression of Types 1 and 2 IL-1*β* Receptors on Monocytes in Unstimulated and LPS-Stimulated MNC Cultures

**Figure 3:**
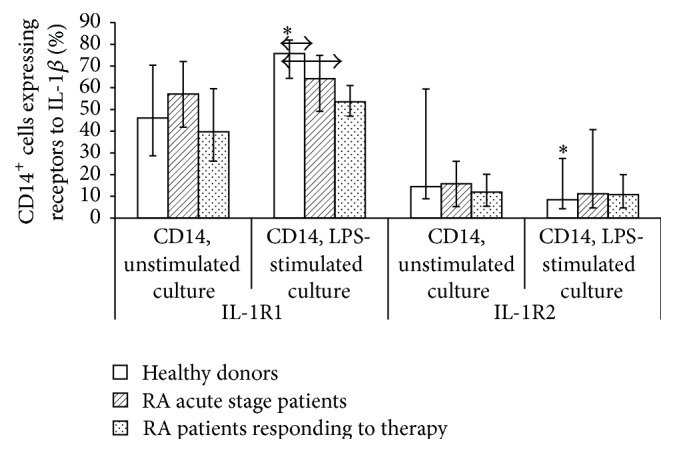
Percentage of CD14^+^ cells expressing IL-1R1 and IL-1R2 in unstimulated and LPS-stimulated MNCs cultured for 24 h derived from healthy individuals (*n* = 150) and RA patients in the acute phase (*n* = 33) and after response to treatment (*n* = 21). The data are presented as the median and interquartile range. The arrows denote significance of intergroup differences and asterisks denote significance of stimulation, *p* < 0.05.

**Figure 4:**
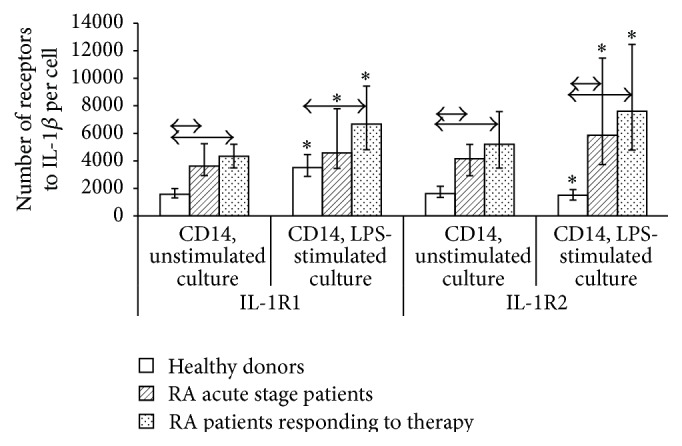
Density of IL-1R1 and IL-1R2 expression on monocytes in unstimulated and LPS-stimulated MNCs cultured for 24 h from healthy individuals (*n* = 150) and RA patients in the acute phase (*n* = 33) and after response to treatment (*n* = 21). The data are presented as the median and interquartile range. The arrows denote significance of intergroup differences and asterisks denote significance of stimulation, *p* < 0.05.

Expression of IL-1 receptors on monocytes (CD14^+^) in unstimulated and LPS-stimulated human PBMC cultures was assessed in a more detailed study of changes in the system of IL-1*β* receptors under a simulated proinflammatory response. To study the ability of cells to respond to stimulation, we assessed the expression of IL-1*β* receptors types 1 and 2 on monocytes of PBMC cultured for 24 h in the presence and absence of LPS (O55 : B5). We determined the percentage of monocytes expressing types 1 and 2 receptors (Figure 3) and calculated the absolute number of receptors per cell (Figure 4).

Culturing in the presence of LPS for 24 h increased the density of IL-1 receptor expression in both healthy individuals and RA patients with higher indices in RA patients compared with healthy volunteers. The stimulation coefficient (quotient of dividing the percentage of cells or the number of receptors in LPS-stimulated culture to that in unstimulated one) for the number of IL-1R1 receptors in RA patients in the acute phase and RA patients who had responded to treatment was consistently lower than that in healthy individuals (1.29 and 1.49 versus 2.19, resp.), while the stimulation coefficient for the number of IL-1R2 receptors was significantly higher (1.52 and 1.43 versus 0.91, resp.). However, consistent LPS-induced changes in the percentage of receptor-carrying cells were observed only for healthy individuals (with an increase for type 1 receptor and a decrease for type 2 receptor), while no response to stimulation was observed in RA patients according to the percentage of positive cells.

When comparing types 1 and type 2 receptors, we found that, unlike intact monocytes (for which the differences were shown only for healthy individuals), the ratio between the number of cells expressing IL-1 receptors of different types in unstimulated and LPS-stimulated cultures changed significantly. The percentage of IL-1R1^+^ monocytes was significantly higher than that of IL-1R2^+^ in all the groups under study. However, the densities of expression of types 1 and 2 receptors in monocytes of healthy individuals in unstimulated cultures were almost identical; stimulation with LPS consistently increased the density of type 1 receptors and reduced the density of type 2 receptors. The average number of IL-1R1 and IL-1R2 receptors on monocytes derived from RA patients was not significantly different.

### 3.3. Serum Levels of IL-1*β*, Soluble Types 1 and 2 IL-1*β* Receptors, and IL-1 Receptor Antagonist

**Figure 5:**
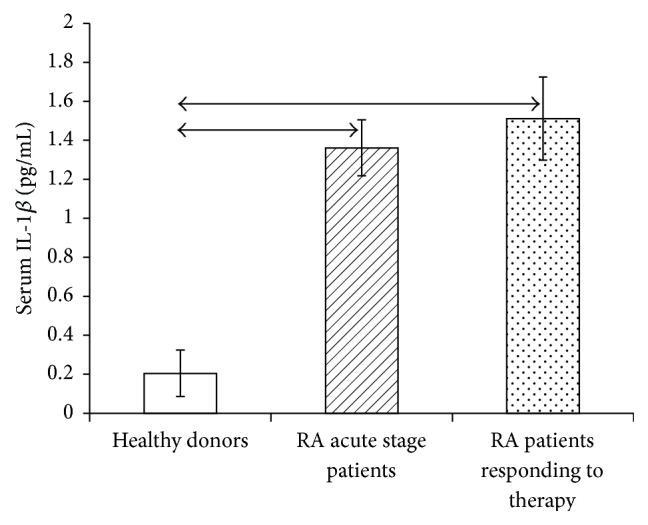
(a) Serum level of IL-1*β* in healthy individuals (*n* = 150) and RA patients in the acute phase (*n* = 28) and after response to treatment (*n* = 25), in pg/mL. The data are presented as the mean ± standard deviation of the mean. The arrows denote significance of intergroup differences, *p* < 0.05.

**Figure 6:**
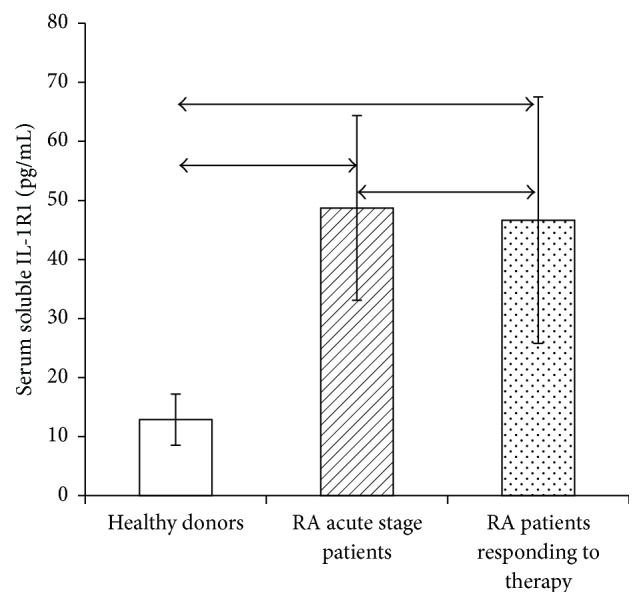
(a) Serum levels of soluble IL-1*β* receptor type 1 in healthy individuals (*n* = 150) and RA patients in the acute phase (*n* = 33) and after response to treatment (*n* = 21), in pg/mL. The data are presented as the mean ± standard deviation of the mean. The arrows denote the significance of intergroup differences, *p* < 0.05.

**Figure 7:**
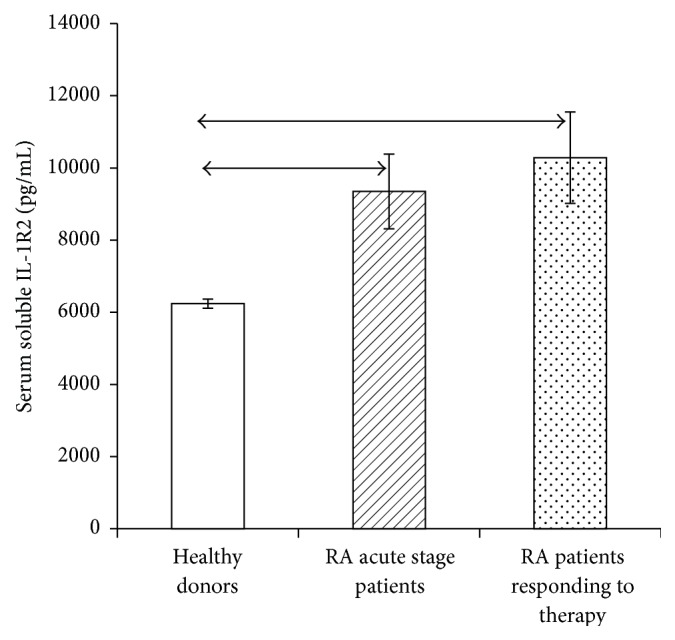
(a) Serum levels of soluble IL-1*β* receptor type 2 in healthy individuals (*n* = 150) and RA patients in the acute phase (*n* = 33) and after response to treatment (*n* = 23), in pg/mL. The data are presented as mean and standard deviation of the mean. The arrows denote the significance of intergroup differences, *p* < 0.05.

Because the effect of IL-1 on cells depends significantly on the relative serum concentration of soluble IL-1*β* receptors and cytokine, the serum contents of IL-1*β* (Figure 5) and soluble IL-1*β* receptors types 1 and 2 (Figures 6 and 7) were studied by ELISA in RA patients in the acute phase and RA patients who responded to treatment.

According to the peripheral blood serum level of the cytokine, the IL-1 level in RA patients was higher than that in healthy individuals and did not decrease after patients had exhibited a clinical response to treatment.

RA patients had higher levels of both types of soluble receptors compared with healthy individuals. Patients who had exhibited a response to therapy had a lower level of type 1 receptor but not type 2, which may be due to the fact that type 2 receptor does not function as a buffer for IL-1.

**Figure 8:**
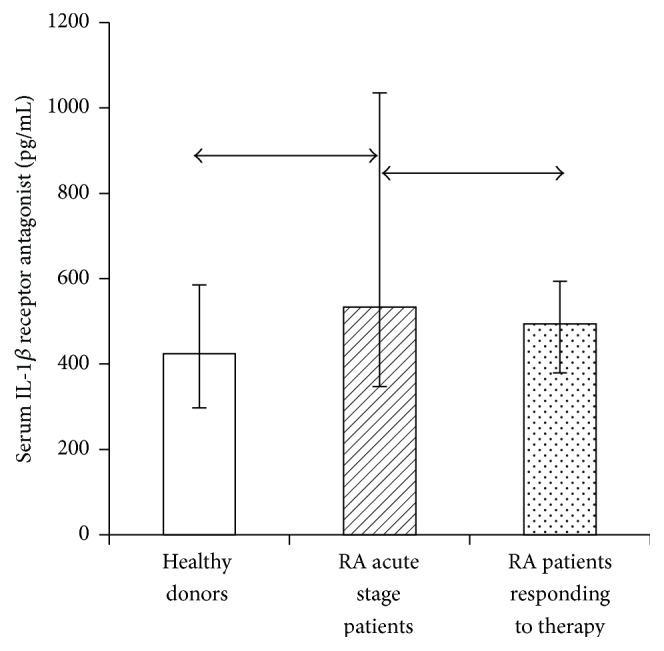
Serum content of IL-1 receptor antagonist in healthy individuals (*n* = 74) and patients with rheumatoid arthritis in the acute phase (*n* = 28) and after response to treatment (*n* = 23), in pg/mL. The data are presented as median and interquartile range. The arrows denote significance of intergroup differences, *p* ≤ 0.05.

Furthermore, the system regulating the biological effects of IL-1*β* also includes IL-1 receptor antagonist, because it competes for binding to cytokine receptors, thus modulating the effect of IL-1*β* on cells. Hence, it was important to assess the serum content of raIL-1 in RA patients in the acute phase and those who had responded to treatment compared with healthy individuals (Figure 8).

RA patients in the acute phase had a significantly increased receptor antagonist level compared with those of healthy individuals and RA patients who had responded to treatment. A positive correlation between mediator level in the acute phase and after effective therapy was detected, suggesting that this indicator can be used as a marker of treatment effectiveness. Also we found several statistically significant correlations between IL-1 receptors parameters and serum contents of soluble mediators.

We established a number of correlations between serum levels of TNF*α* and its soluble receptors and parameters of IL-1 receptors expression in PBMC culture. Particularly content of TNF*α* in RA patients in acute stage negatively correlated (*r* = −0.43, *p* < 0.05) with the percentage of intact IL-1R2^+^ monocytes and had no correlations with any parameter of receptor expression in the cultures. However, in RA patients responding to therapy content of TNF*α* positively correlated (*r* = +0.45, *p* < 0.05) with the percentage of IL-1R2^+^ monocytes in LPS-stimulated cultures and negatively (*r* = −0.48, *p* < 0.05) with the number of receptors on these cells.

## 4. Discussion

The proinflammatory cytokine IL-1*β* has a broad range of effects on the development and course of RA through its receptors, at both the local and systemic levels. However, at the molecular level these effects are regulated by mediator production and changes in the systems of signal transmission to immunocompetent cells or its blockade. The role of IL-1*β*, raIL-1, and soluble IL-1 receptors in this pathology has been demonstrated previously [24–26]. However, the changes in the expression of membrane-bound receptors have been insufficiently characterized.

IL-1*β* exhibits its proinflammatory effect only after binding to specific membrane-bound type 1 receptors, while type 2 receptors act as decoy receptors. Therefore, the balance between different types of soluble and membrane-bound receptors will determine whether a cell responds to IL-1 or not and variability in intracellular effects. Additionally, the biological effects of cytokines depend on the concentration of their soluble forms both due to binding to the membrane forms of receptors and due to induction of expression and/or shedding of cytokine receptors. Hence, the comprehensive assessment and comparison of the levels of soluble factors regulating cytokine activity and expression of membrane-bound forms of receptors enable a richer and more accurate understanding of the system regulating the biological effects of cytokines. One should bear in mind that changes in density of receptor expression on the cell surface are a key mechanism of regulation of the biological properties of cytokines [27–29].

However, cytokine function can be minimal if receptor expression is downregulated. In the case of excessive expression of receptors, cell populations will respond actively to the ligand-receptor interaction. Moreover, it has been demonstrated for some immune mediators that there is a certain threshold level of expression density, which switches the signaling pathways between the fundamentally different functions [30, 31]. Thus, it is insufficient to only know the percentage of positive cells in a subpopulation when studying the membrane forms of receptors. One also needs to determine the density of receptor expression on the cell surface in standardized units that are independent of the equipment or settings used (as opposed to the commonly employed direct measures of fluorescence intensity given in terms of arbitrary units). Hence, we used calibration particles (BD Biosciences) to convert the units of mean fluorescence intensity (MFI) to the number of receptor molecules on the cell surface. Tables 1 and 2 summarize the data on differences obtained for RA patients in the acute phase and after they had responded to therapy, as compared with healthy individuals, as well as the differences between the phases of the disease.

We demonstrated that intact PBMCs differ both in terms of the relative percentage of cells expressing IL-1*β* receptors types 1 and 2 and in terms of the absolute number of receptors on them. The high density of receptor expression on a cell does not always imply a high percentage of positive cells, and* vice versa*. Moreover, increased or reduced density of receptor expression on cells in pathology compared with normal does not always correlate with the percentage of cells carrying these receptors. It was demonstrated for the expression of IL-1*β* receptors type 1 that the percentage of IL-1R1^+^ cells increases while the density of expression of these receptors on these cells decreases simultaneously in B cells of patients who had responded to treatment. An opposite ratio is observed in monocytes of RA patients regardless of the stage of the disease (i.e., reduced percentage of IL-1R1^+^ cells with a simultaneous increase in density). This feature was also revealed in LPS-stimulated monocytes. Thus, the opposing changes in the percentage of cells and density of expression of receptor in these subpopulations of immunocompetent cells can act as a mechanism for cytokine binding to target receptors on specific subpopulations.

The changes in the percentage of receptor-expressing cells in subpopulations or the changes in density of receptor expression in pathology compared with health are caused by different mechanisms. It was demonstrated for IL-1*β* that upregulated expression of IL-1R2 on cells may either cause the absence of cellular response to IL-1 [32, 33] or reduce the cytokine effect on cells. The number of type 2 decoy receptors on B lymphocytes is reduced in RA patients in the acute phase, but it is compensated for by the higher percentage of IL-1R2^+^  В cells in patients who had a clinical response to treatment as compared to with healthy individuals. Furthermore, the percentage of IL-1R2^+^ T cells increased significantly in patients who had responded to therapy, while upregulation of expression of these receptors was observed in monocytes in RA patients regardless of the phase of the disease. Thus, these changes demonstrate that IL-1R2 plays a crucial role in cellular response to treatment in RA patients. A hypothesis can be put forward that this type of membrane-bound receptor could be targeted in future therapeutic approaches in rheumatoid arthritis.

According to the changes in function of membrane-bound receptors and their competition for binding to a corresponding cytokine, the balance between the two receptor types is a key factor showing the status of the system regulating the biological effects of cytokines. The changes in the percentage of receptor-carrying cells in a subpopulation, the changes in mean density of expression of types 1 and 2 receptors, and the changes in this balance in pathology compared with health provide the two alternative mechanisms of modulation of cytokine effect to increase its chances for binding to a certain receptor type on cells of a certain subpopulation. We demonstrated that the percentage of cells expressing IL-1R1 is higher than the percentage of IL-1R2^+^ cells among T and B cells and monocytes only in healthy donors; after the cells are cultured, this percentage is higher among monocytes in all the groups under study. In terms of the number of receptors on cells of intact subpopulations in healthy individuals, T and B cells are characterized by upregulated expression of type 1 receptors, while monocytes are characterized by upregulated expression of type 2 receptors. However, this balance is disturbed in RA patients and there are changes between the mean number of types 1 and 2 receptors in neither subpopulation. Thus, the balance between different types of cytokine receptors having different functions is changed in pathology, which explains the disturbance in regulation of effects of proinflammatory mediators in rheumatoid arthritis. In addition, for a more detailed consideration and establishment of causes and effects of these differences also the relationship with other components of cytokine network and regulatory proteins (such other proinflammatory cytokines and IL1RAcP) need to be considered.

The detected differences in intact cells and in the monocyte response to activation by LPS demonstrate that monocytes can regulate the activity of IL-1*β* both by changing the percentage of positive cells or the number of receptors per cell and by simultaneously changing these two indicators in opposite directions.

Soluble receptors are powerful regulators of cytokine activity as they can neutralize IL-1*β* in the systemic circulation [15, 20] and cytokine complexed with soluble receptors can have a buffering function [21]. However, certain differences in implementation of their functions have been demonstrated for different types of soluble receptors. Soluble IL-1R2 is characterized by higher binding affinity compared with the corresponding type 1 receptors (i.e., they are major contributors to inhibition of cytokine activity). sIL-1R1 has a better buffering function and is simultaneously characterized by higher affinity to the IL-1 receptor antagonist and IL-1*α*, rather than IL-1*β*. Furthermore, it was shown for IL-1*β* that there exists a specific receptor antagonist competing with cytokine for binding to receptors. We demonstrated that the serum levels of IL-1R2 in all the groups under study were significantly higher than those of type 1 receptors. However, RA patients in the acute phase had higher levels of type 1 receptors compared with healthy individuals. This indicates that adaptability of the organism to inflammation is reduced. We revealed the correlations between parameters of soluble mediators in RA patients who had responded to treatment and those in the same patients in the acute phase (reduced serum levels of IL-1R1 and raIL-1), which can be used to attest to treatment effectiveness.

## 5. Conclusions

The resulting data are indicative of differences in expression of IL-1*β* receptors in various subpopulations of immunocompetent cells in normal cells and in pathology. Additionally, they show changes both in indicators of mediator production accompanying inflammatory pathologies and in the system of receptor regulation on the cell surface. Therefore, it is important to determine both the relative percentage of cells expressing receptors to immunomodulatory cytokines and the levels of membrane-bound receptors, because the density of expression is characterized by disease-induced changes that cannot be detected when assessing the percentage of positive cells. It was found that oppositely directed changes in the density of expression of IL-1*β* receptors type 1 and in the percentage of IL-1R1^+^ cells in B cells and monocytes take place, thus resulting in different mechanisms of regulation of cellular response to cytokines via changing the parameters of expression of different types of receptors.

## Figures and Tables

**Table 1:** Changes in parameters of expression of membrane-bound forms of IL-1*β* receptors types 1 and 2 on intact cells and monocytes in spontaneous and LPS-stimulated 24 h cultures and in serum levels of IL-1*β* cytokine and soluble receptors types 1 and 2 in healthy individuals and in patients with rheumatoid arthritis in the acute phase (*n* = 40) and after response to treatment (*n* = 24). The arrows denote the changes (↑ = increased, ↓ = decreased) compared with healthy individuals. % shows the changes in the percentage of positive cells; *ρ* is the change in density of receptor expression on cells. *∗* = a significant (*p* < 0.05) decrease in indicator in the group of patients who responded to therapy compared with this indicator in RA patients in the acute phase.

	RA patients in acute phase		RA patients after response to treatment	
	IL-1R1	IL-1R2	IL-1R1	IL-1R2
IL-1R-expression in cell subsets				
T cells	↓ *ρ*		↓ *ρ*	↑ %
B cells	↓ *ρ*	↓ *ρ*	↑ % and ↓ *ρ*	↑ %
Monocytes	↓ % and ↑ *ρ*	↑ *ρ*	↓ %^*∗*^ and ↑ *ρ*	↑ *ρ*
Monocytes in spontaneous PBMC cultures	↑ *ρ*	↑ *ρ*	↑ *ρ*	↑ *ρ*
Monocytes in LPS-stimulated PBMC cultures	↓ % and ↑ *ρ*	↑ *ρ*	↓ % and ↑ *ρ*	↑ *ρ*

Serum content				
IL-1*β*	↑		↑	
raIL-1	↑	↑	*∗*	*∗*
Soluble receptor	↑	↑	↑^*∗*^	↑

**Table 2:** Balance between IL-1*β* receptors types 1 and 2 in terms of expression of membrane-bound forms of receptors on intact cells and monocytes in 24 h spontaneous and LPS-stimulated PBMC cultures in healthy individuals and in patients with rheumatoid arthritis in the acute phase (*n* = 33) and after response to treatment (*n* = 23). Each table cell indicates the more highly expressed receptor for a selected subpopulation in a studied group. “=” denotes that no significant difference in receptor expression was observed.

	In terms of percentage of cells			In terms of the number of receptors		
	Healthy individuals	RA patients in acute phase	RA patients after response to treatment	Healthy individuals	RA patients in acute phase	RA patients after response to treatment
T cells	IL-1R1	=	=	IL-1R1	=	=
B cells	IL-1R1	=	=	IL-1R1	=	=
Monocytes	IL-1R1	=	=	IL-1R2	=	=
Monocytes in spontaneous cultures	IL-1R1	IL-1R1	IL-1R1	=	=	=
Monocytes in LPS-stimulated cultures	IL-1R1	IL-1R1	IL-1R1	IL-1R1	=	=
